# Liquid Biopsy in Diagnosis and Prognosis of High-Grade Gliomas; State-of-the-Art and Literature Review

**DOI:** 10.3390/life12030407

**Published:** 2022-03-11

**Authors:** Lapo Bonosi, Gianluca Ferini, Giuseppe Roberto Giammalva, Umberto Emanuele Benigno, Massimiliano Porzio, Evier Andrea Giovannini, Sofia Musso, Rosa Maria Gerardi, Lara Brunasso, Roberta Costanzo, Federica Paolini, Francesca Graziano, Gianluca Scalia, Giuseppe Emmanuele Umana, Rina Di Bonaventura, Carmelo Lucio Sturiale, Domenico Gerardo Iacopino, Rosario Maugeri

**Affiliations:** 1 Neurosurgical Clinic, AOUP “Paolo Giaccone”, Post Graduate Residency Program in Neurologic Surgery, Department of Biomedicine Neurosciences and Advanced Diagnostics, School of Medicine, University of Palermo, 90127 Palermo, Italy; robertogiammalva@live.it (G.R.G.); umberto.emanuele.benigno@gmail.com (U.E.B.); massimiliano.porzio1@gmail.com (M.P.); evier.andrea.giovannini@gmail.com (E.A.G.); sofiamusso.sm@gmail.com (S.M.); rosamariagerardimd@gmail.com (R.M.G.); brunassolara@gmail.com (L.B.); robertacostanzo3@gmail.com (R.C.); federicapaolini94@gmail.com (F.P.); gerardo.iacopino@gmail.com (D.G.I.); rosario.maugeri1977@gmail.com (R.M.); 2 Department of Radiation Oncology, REM Radioterapia srl, 95125 Catania, Italy; gianluca.ferini@grupposamed.com; 3 Unit of Neurosurgery, Garibaldi Hospital, 95124 Catania, Italy; fragraziano9@gmail.com (F.G.); gianluca.scalia@outlook.it (G.S.); 4 Trauma Center, Gamma Knife Center, Department of Neurosurgery, Cannizzaro Hospital, 95125 Catania, Italy; umana.nch@gmail.com; 5 Department of Neurosurgery, Fondazione Policlinico Universitario “A. Gemelli” IRCCS, Università Cattolica del Sacro Cuore, 00168 Rome, Italy; rina.di.bonaventura@hotmail.it (R.D.B.); cropcircle.2000@virgilio.it (C.L.S.)

**Keywords:** liquid biopsy, high-grade glioma, GBM, circulating tumor DNA, extracellular vesicles, miRNA, next-generation sequencing

## Abstract

Gliomas, particularly high-grade gliomas, represent the most common and aggressive tumors of the CNS and are still burdened by high mortality and a very poor prognosis, regardless of the type of therapy. Their diagnosis and monitoring rely on imaging techniques and direct biopsy of the pathological tissue; however, both procedures have inherent limitations. To address these limitations, liquid biopsies have been proposed in this field. They could represent an innovative tool that could help clinicians in the early diagnosis, monitoring, and prognosis of these tumors. Furthermore, the rapid development of next-generation sequencing (NGS) technologies has led to a significant reduction in sequencing cost, with improved accuracy, providing a molecular profile of cancer and leading to better survival results and less disease burden. This paper focuses on the current clinical application of liquid biopsy in the early diagnosis and prognosis of cancer, introduces NGS-related methods, reviews recent progress, and summarizes challenges and future perspectives.

## 1. Introduction

Gliomas are the most common malignant tumor of the CNS. Their grade of malignancy varies across histotypes, ranging from grade I to IV [1]. Glioblastoma Multiforme (GBM) is considered the most frequent and aggressive subtype since it is generally associated with substantial mortality and morbidity. Indeed, the median Overall Survival (OS) does not exceed 15 months from the diagnosis, following the standard therapeutic protocol, consisting of gross total resection followed by adjuvant radiotherapy plus chemotherapy [2,3].

Recent improvements in the field of human genome sequencing have made possible the study of the genetic alterations at the base of these tumors, leading researchers to point out some of the molecular pathways that characterize the genesis, differentiation, and progression of primitive brain tumors. Moreover, tumors which had previously been included in the same diagnostic categories due to macroscopical and histopathological similarities show different and distinctive gene expression profiles and other biological behaviors [4].

Consequently, molecular parameters were introduced in the 2016 WHO classification to define new diagnostic categories of gliomas whose prognoses and therapeutic targets differ from prior varieties [5].

**Figure 1:**
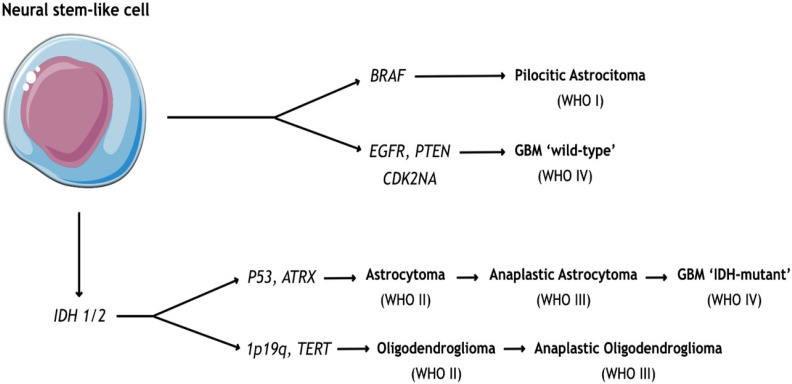
Scheme of gliomagenesis with the most-studied mutations.

As a result of recent progress, it has been demonstrated that glial tumors originate from undifferentiated precursors or neural stem-like cells. These represent a self-renewing cellular pool, concentrated mainly in the subventricular zone and subject to a series of genetic alterations that ultimately lead to ‘gliomagenesis’ [6,7] (Figure 1).

Mutations and amplifications on EGFR genes, for instance, lead to increased tyrosine kinase receptor activity, which is positively involved in cellular proliferation, invasiveness, and differentiation [8]. Also, dysregulation of P53 and RB—induced by internal and external stress signals—causes the alteration of multiple pathways embracing cell metabolism, senescence, and DNA-repair mechanisms [4]. Telomerase aberrations, including ATRX and TERT, are frequently encountered as well [7].

Moreover, glial tumors may show IDH1 mutations or not. In the latter case, they are deemed ‘wild type’ and are associated with a poorer prognosis [4].

In addition, MGMT-promoter methylation can occur, causing the epigenetic silencing of the methylated-DNA-protein-cysteine methyltransferase. The presence of this alteration predicts a subject’s chemosensitivity to temozolomide (TMZ) therapeutic effects.

Non-coding genetic sequences also play a notable role. It has been proven that several types of miRNAs may determine up- or down-regulation of disparate oncogene pools involved in gliomagenesis through an epigenetic modulation [9].

Finally, 1p19q co-deletion is frequently associated with the oligodendroglial phenotype. It is also mutually exclusive to the P53 mutation and the EGFR amplification, which primarily characterize astrocytomas [10].

Generally, there is a strong correlation between the clinical features of the tumor itself, namely the histopathological framing and the prognostic assessment, and the occurrence of the aforementioned molecular mutations has been proven. Therefore, the markers identification stage is crucial for decision-making in glioma treatment, as it can predict the therapeutic responses for those patients with histopathologically similar tumors.

These markers are analyzed in daily clinical practice using immunohistochemical and molecular biology techniques. These methods start from tissue samples that do not fully include the spatial and temporal heterogeneity of the whole tumor. Furthermore, collecting these samples often requires a complex surgical procedure, with substantial risks for the patient.

Liquid biopsy does not require a sample of tumoral tissue. It provides the opportunity to detect, analyze, and monitor cancer in various biologic samples such as blood, CSF, or urine [11,12].

Tissue biopsies are usually taken from the primary tumor and reflect its molecular composition at tissue sampling. Due to the heterogeneity of brain tumors, there is a chance that some of their characteristics remain undetected. Indeed, a blood biopsy can reflect the biochemical changes acquired during blood-borne dissemination, which may not be present in the primary tumor [13,14].

Moreover, liquid biopsy is not aggravated by risks of bleeding, damage in eloquent areas, or neurological deficits related to surgery. It can always be performed without risk for the patient, regardless of the lesion site, and there is no limitation to the number of samples taken [12,15].

New gene sequencing techniques, such as NGS, play a fundamental role in this context. Two methods are currently used for nucleotide sequencing: polymerase chain reaction (PCR)-based sequencing and next-generation sequencing (NGS) [15].

Mutation analyses based on qPCR are well established in clinical practice. Well-designed qPCR assays are very sensitive. However, qPCR assays are target-specific, so they cannot detect mutations that they were not initially designed for. Next-generation sequencing (NGS) is a high-throughput technology that enables multiple gene testing, designed to overcome the Sanger sequencing limitations. Today, this technology has evolved to be used in almost all areas of genomic research (sequencing of DNA, RNA, miRNA, ChIP, and methylation) [16,17].

**Figure 2:**
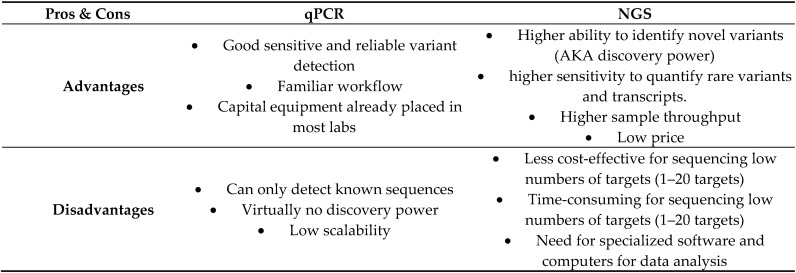
Advantages and disadvantages of quantitative polymerase chain reaction (qPCR) versus next-generation sequencing (NGS).

The main difference of NGS is a more-excellent discovery capability than that of qPCR. NGS platforms have made DNA sequencing dramatically more straightforward and faster. From a quantitative perspective, while the Sanger method could sequence 10^5^ base pairs per run, the current highest throughput commercial instruments can generate nearly one terabase per run (10^12^ base pairs). It allows for the sequencing of all the coding exons of the genome (whole exome sequencing, WES) and even the sequencing of a complete genome (whole genome sequencing, WGS) in a shorter time and at an affordable price [18]. The choice between NGS and qPCR depends on several factors, including the number of samples, the total amount of sequence in the target regions, budgetary considerations, and study goals. qPCR, for instance, could be a good choice when the number of target regions is low. On the other hand, NGS allows a better allocation of time and resources than PCR-based technologies due to the possibility of sequencing multiple genes across multiple samples simultaneously (Figure 2).

Hence, the need to develop new monitoring methods seems mandatory, and liquid biopsy and next-generation sequencing could represent a step forward in diagnosing and managing HGGs.

## 2. Materials and Methods

A systematic literature search was performed on the PubMed database without time limits according to PRISMA guidelines [19]. The research strategy initially relied on title and abstract analysis. If a title and abstract met the inclusion criteria, then the article’s full text was retrieved for further investigation. Two authors (S.M. and U.E.B.) independently assessed eligibility, and differences were resolved with the help of a third author (L.B). This process also permitted the assessment of the study’s risk of bias. The data collection process was conducted without using any automated tools. No ethical approval was required for this study. The following medical subject headings (MeSH) and free-text terms were combined: “liquid biopsy”, “circulating tumor DNA” AND “high-grade glioma”, “HGG”, “glioblastoma”.

**Figure 3:**
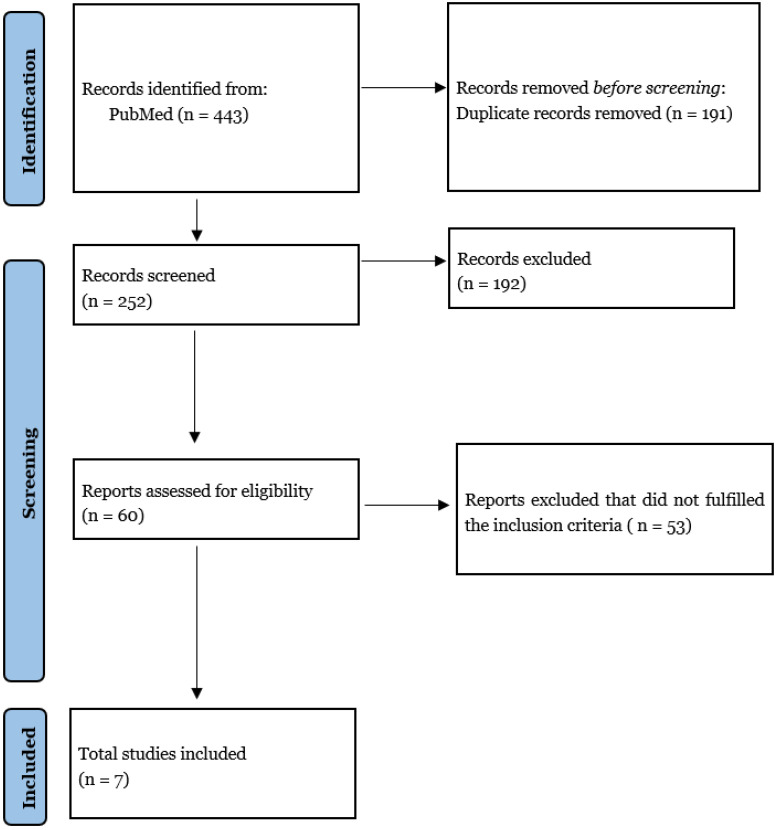
PRISMA 2020 Flow diagram.

Details of the search strategy are shown in Figure 3.

**Eligibility criteria**

The articles were selected according to the following inclusion criteria:

- Full articles in English
- Studies already in the “clinical phase”
- Studies regarding patients with only high-grade gliomas
- Studies regarding patients with age > 18
- Studies reporting data in terms of sensitivity and specificity.

The studies selected are shown in Table 1.

## 3. Results

Selected studies

From the whole literature review, 60 studies were identified, of which only 7 were included for this systematic review, based upon the a priori-defined inclusion criteria, according to PRISMA (Preferred Reporting Items for Systematic Reviews and Meta-Analyses) flow diagram (Figure 3).

After the full-text screening, seven articles were included in the present study, which is primarily heterogeneous in terms of biomarkers evaluated: among them, one focused on the evaluation of ctDNA, four on the role of miRNAs, and two on the part of extracellular vesicles. All of these are prospective studies.

The characteristics researched in all of the articles are shown in Table 1; these are: the number of patients enrolled, the biomarkers evaluated, the detection method used for each one, the sampled fluid, the role of biomarkers (diagnostic/prognostic/monitoring), and their sensitivity and specificity.

Among all the articles assessed for eligibility, we selected only the papers that expressed the usefulness of the selected biomarkers in terms of sensitivity and specificity: One author (S.M.) extracted data from included articles. Extracted data were then confirmed by an additional author (L.B).

### 3.1. Biomarkers

#### 3.1.1. miRNAs

Most of the selected studies focused on miRNAs. They are non-coding RNA molecules that influence gene expression. Evaluated miRNAs were: miR-21, miR-222, miR-124-3p, miR-17-5p, miR-125b, miR-221, and miR-100. In all the studies, the detection method was PCR performed on blood samples. Three studies considered different kinds of miRNAs able to diagnose glioma patients with variable sensitivity and specificity, and three studies attributed to miRNAs a prognostic role.

MiR-21, miR-222, and miR-124-3p could be used as useful molecular biomarkers for complementing clinical evaluations of early tumor progression during post-surgical therapy in patients with HGG; upregulation of exosomal miRNAs is associated with tumor relapse.

It has been demonstrated that serum miR-100 expression levels were downregulated in GBM compared to the healthy controls. Furthermore, serum miR-100 levels significantly increased following treatment; this may indicate that serum miR-100 levels can differentiate between GBM patients and healthy controls, and it might be sensitive to treatment response.

The clinical role of miR-221 and miR-222 in GBM diagnosis and patient survival has also been investigated with high sensitivity and specificity. Another study investigated the role of miR-17-5p, miR-125b, and miR-221: the assessment of these miRNA’s expression has been proven helpful in the diagnosis and prognosis of patients primarily related to the response to treatment.

#### 3.1.2. ctDNA

The study evaluated the circulating tumor DNA (ctDNA), using plasma as a fluid sample. It has been suggested that the integrated analysis of cfDNA plasma concentration, gene mutations, and gene–gene fusions can serve as a diagnostic modality for distinguishing GBM patients who may benefit from targeted therapy, with both good sensibility and good specificity (80% and 90%, respectively) [22]. It is also the only study in which the detection method was based on the NGS technique.

#### 3.1.3. Extracellular Vesicles

Two articles assessed the role of extracellular vesicles as biomarkers for high-grade gliomas through the evaluation of EGFRvIII status. Manda SV et al. [25] established a serum-based detection method for EGFRvIII in high-grade gliomas that may serve as an optimal noninvasive diagnostic method. Figueroa et al. [26] demonstrated that CSF-derived EVs in glioblastoma patients contain an RNA signature, reflecting the wtEGFR expression and EGFRvIII status. The typical serum volume used for RNA isolation was around 2–3 mL per sample in the first study, and 1 mL in the second.

## 4. Discussion

Currently, GBM still represents a therapeutic challenge despite advancements in the tools and techniques for surgical resection [27,28]. Thus, novel therapies are needed to personalize the treatment of high-grade gliomas. In this context, an accurate, reliable, repeatable, and non-invasive tool is necessary to characterize tumor features.

However, the lack of valuable biomarkers hinders the clinical management of glioma patients. Liquid biopsies may represent a reliable and non-invasive technique [29].

Liquid biopsy aims to avoid the need for surgery to obtain a biopsic sample, determine a classification of non-surgical lesions, make prognostication before therapies, and maximize treatment effectiveness.

### 4.1. EVs (Extracellular Vesicles)

Among the biomolecular markers employed for liquid biopsy, EVs (extracellular vesicles) appear promising as a means to revolutionize GBM screening, therapy, and patient outcome. It has been proven that GBM produces a high number of EVs, which can cross the BBB; moreover, EVs differ from vesicles of normal glial cells, and they can thus be detected by CD9 as a recognition marker [30]. Osti et al. [31] showed increased EV concentration in the peripheral blood of GBM patients compared to healthy controls. This kind of EV also differs from patients affected by brain metastases and extra-axial brain tumors. EVs reflect molecular organization and tumoral microenvironment, and they are involved in many tumor activities, such as tumor proliferation, metabolic activity, angiogenesis, immune surveillance, and drug resistance [32].

The primary biological fluids used to detect the EVs are blood and CFS. Between the two, CSF has a higher EV concentration than blood; therefore, CSF samples show higher specificity. Nevertheless, CSF sampling is an invasive and not easily repeatable method related to potential complications. On the other hand, a blood sample is easier to obtain and can be repeated several times, but detection is more difficult given the complexity of its composition [33].

EVs appear to be promising in clinical practice thanks to their non-invasive sampling method and their multilayered informative power: they are cellular products of the primary tumor. They can depict the status of tumor evolution better than ctDNA [33]. Moreover, exosomal DNA shows better sensitivity and specificity than ctDNA in detecting mutational frequency and could be an informative tool for GBM diagnosis and follow-up [33].

Interestingly, EVs could potentially be used to detect the chemoradiation therapy (CRT)-responder patient, distinguishing between pseudo- and actual progression on radiological imaging after CRT, thus helping to obtain the best therapeutic management [34].

### 4.2. CTCs (Circulating Tumor Cells) and ctDNA (Circulating Tumor DNA)

CTCs (circulating tumor cells) are rare neoplastic cells that spread from primary tumors into biofluids [35]. CTCs can cross the BBB, entering the circulating blood flow, thus allowing their detection in the blood sample. CTC screening can be obtained through several techniques, such as telomerase assay, antibodies, and micro-fluid devices [36], which show high variability in recognizing circulating tumor cells.

The liquid biopsy aimed at detecting CTCs could represent a non-invasive technique for optimizing cancer care and advancing personalized medicine [37].

CTCs share some characteristics with the primary tumor, such as mutations and gene expression signatures (e.g., EGFR). Because of these characteristics, CTCs may reasonably be investigated so as to define their role in tumor progression and their potential role for tailoring a patient-specific therapy [38].

ctDNA is a subset of circulating free DNA (cfDNA) produced by CTC apoptosis. It has been used for CNS tumor detection and prognostication to identify genomic alterations and epigenetic signatures.

The techniques mainly used for ctDNA screening are PCR and NGS. PCR is a low-cost, simple, and highly specific technique, but it shows a low sensitivity in CTC detection.

Because of the low amount of ctDNA in the bloodstream, CSF shows higher sensitivity in detecting GBM CTCs [39]. Nevertheless, a recent study developed a glioma epigenetic liquid biopsy score called GeLB [40]; this score is capable of distinguishing glioma patients (GeLB > 50%) with high specificity and sensibility. Because of this capability, GeLB could be useful in early diagnosis and [39] personalized tumor evaluation [41].

As regards epigenomic signatures such as DNA methylation, a more comprehensive range of applications is available. This method is not limited to known alterations, but it can identify highly specific tissues, thus facilitating the diagnosis of CNS tumors [33,42]. Moreover, it has been proven that methylation status is associated with therapeutic response and prognosis in GBM patients, revealing this technique as a reliable tool for personalized therapies.

ctDNA can also detect IDH1 mutation status with high sensitivity and specificity [38]. Regarding the MGMT promoter methylation, the literature has shown that a CSF sample is more suitable than serum because of the higher rate of sensibility [43].

Even if it has been previously documented that ctDNA mutations may be found only in 10% of GBM patients, sensitivity has increased up to 55% in the latest studies [44]. Furthermore, it has been demonstrated that ctDNA could pass through the BBB in half of the patients with the potentially targetable genomic alteration. Evidence in the literature suggests that it is possible to perform a ctDNA analysis for GBM genomic profiling before an invasive surgical biopsy or in patients that could not undergo surgery [45].

Interestingly, ctDNA could be used for an off-label target therapy during follow-up and to monitor disease progression. In addition, ctDNA may be effectively used to differentiate high-grade [46,47] from low-grade gliomas. However, ctDNA potential in therapeutic planning is still limited.

### 4.3. miRNAs

The deregulation of miRNA expression plays a critical role in cancer genesis. The detection of miRNAs in biofluids has shown significant advantages compared to mRNAs, which go through degradation very early and are not easily storable. The evaluation of miRNA has been mainly performed by PCR, which is more expensive than microarray methods, but it shows higher sensitivity. Several miRNAs have been identified as potential tumor biomarkers for tumor diagnosis and prognosis [48].

MiR-21 is strictly related to GBM as a clinical biomarker among the different miRNAs. Several studies have demonstrated the up-regulation of miR-21 and the down-regulation of miR342-3p in GBM patients’ serum. The up-regulation of miR-21 seems related to a worse prognosis in these patients in terms of overall survival (OS), higher tumor grade, and radio-resistance [41]. Literature also suggests that the serum level of circulating miR-21 decreases after tumor resection. For this reason, miR-21 can be used as a predictive biomarker in response to therapy.

Among biofluids, plasma is more reliable for miRNA detection because of the possible coagulation of serum that can alter the existing repertoire of miRNAs. CSF has also been used to detect miRNA, especially miR-21, which seems to be related to high-grade gliomas. In conclusion, CSF represents a more sensitive sample for identifying miRNA, diagnosing HGG, and discriminating GBM from brain metastasis; however, further studies are needed.

### 4.4. TEPs

Tumor-educated platelets (TEPs) are circulating carriers for protumor factors and have a role in determining tumor progression and resistance to therapies [49]. Di Vito et al. [50] hypothesized that the hypervascularization caused by GBM recruits many platelets (PLTs), which leads to the activation and release of pro-angiogenic factors, such as VEGF and PDGF. It has also been demonstrated to increase VEGF and PDGF in GBM patients. The direct consequence of this phenomenon is the increase of Sphingosine 1-Phosphate (S1P) into PLTs: they can be released, causing high expression of VEGF and thus promoting neoangiogenesis. This phenomenon should be considered for bevacizumab personalized therapy [51].

Up until now, the clinical relevance of TEPs has remained unclear.

### 4.5. Liquid Biopsy Limits in Clinical Uses, Diagnosis, Prognosis

In recent years, enormous progress has been made in the development of diagnostic and prognostic devices based on NGS or PCR technologies (i.e., Guardant360^®^ by GuardantHealth, AmoyDx^®^ by Amoy Diagnostics, and Signatera™ by Natera) and in their applicability in clinical practice. These tools have been used for non-CNS solid tumors with excellent sensitivity and specificity [52,53]. To date, the Signatera™ assay is used after surgery to evaluate molecular residual disease (MRD) in patients with colorectal cancer and the consequent need for adjuvant chemotherapy to personalize the medical treatment [54]. On the other hand, the FDA-approved Guardant360^®^ test has been demonstrated to identify guideline-recommended biomarkers in all patients with newly diagnosed metastatic non-small cell lung cancer (mNSCLS), at a rate at least as high as that of standard-of-care tissue genotyping, with increased tissue concordance, more rapidly and completely than tissue-based genotyping [55]. Finally, Resolution Bioscience ctCDx™ has demonstrated its capacity to improve access in a clinically relevant turnaround time (TAT) for oncology patients with an excellent cost/time ratio [56].

These current assays can improve personalized and patient-specific medicine, shed light on the complex mechanisms of tumor genesis and progression, and develop highly targeted therapies [57].

However, despite technological development, several elements represent a limitation to introducing liquid biopsy in the clinical routine for detecting and monitoring CNS tumors. First of all, the BBB reduces the amount of detectable material in the bloodstream, and the high heterogeneity of GBM makes it challenging to describe tumor features accurately [30]. Tissue sampling represents another issue: both blood and CSF samples have intrinsic advantages and disadvantages that should be considered in the diagnostic and therapeutic workup.

Even if they have been considered the future of GBM characterization, EVs are secreted from tumor GBM cells and non-malignant cells; therefore, broader comprehension of their role is needed, together with standardization in EV sampling and evaluation [30]. Furthermore, the more suitable biofluid for EV detection is still debated. In particular, CSF does not show high sensitivity, with an increased number of false negatives. Clinical use of CTCs is severely limited for many reasons: the techniques of isolation and enrichment are complicated by the low concentration of CTCs in biofluids; the lack of numerous data sets; the complexity and the duration of the procedure; the short life of CTCs in the bloodstream; and the lack of studies enrolling a large number of patients.

Regarding ctDNAs, gliomas seem to be associated with the lowest amount of cfDNAs detectable, and every single molecular analyte has its challenges. Because of their low concentrations, ctDNA detection shows high specificity but low sensitivity, which leads to low false-positive and high false-negative rates. This can be considered a limitation of this detection method, particularly during the early stages when levels of ctDNA are low. Furthermore, ctDNA does not cross the BBB. Another limitation in the employment of ctDNA in clinical practice is the lack of numerous cohorts of patients in the literature and the heterogeneity of tumors studied in scientific works. Unfortunately, there is still no validation of the role of ctDNAs in diagnosis and follow-up for GBM patients.

The usefulness of miRNAs is restricted due to the limitations in standardizing preanalytical features, analytical techniques, and inconsistency in study design and tumor samples [58]. Furthermore, tumor heterogeneity and subtypes could lead to inhomogeneity among different studies. Consequently, miRNAs have failed to enter the clinical practice, given the inconsistency and the irreproducibility of these findings [33,34].

### 4.6. The State of the Art

We have reviewed the current literature about biomolecular features tested in liquid biopsies. Many possibilities that could help in the clinical evaluation and classification of gliomas have come to light.

The techniques evaluated in the present study differ in sensitivity, specificity, and clinical use, but each method focuses on a different aspect of the complexity of tumor progression. According to our results, the actual limitations restrict the implementation of liquid biopsy in clinical practice; therefore, liquid biopsy still cannot be used as the best choice in identifying and classifying CNS tumors.

The necessity of obtaining additional information about high-grade gliomas is increasingly evident [59]. Several studies have concluded that, given the actual state of the art of biomarkers, liquid biopsy is still not ready to be used in clinical practice.

Liquid biopsy aims to find glioma-derived circulating cells and tumor-specific genomic alteration in the bloodstream, as biomarkers detected in biofluids can reflect the local and systemic response to tumor activity.

Furthermore, panels of biomarkers should be used, rather than single markers affected by lower accuracy. Remarkably, the more severe the tumor malignancy, the more biomarkers are dysregulated; thus, liquid biopsy can help detect glioma grade through multi-biosources and multi-biomolecule-based blood tests.

The main limitations of this technique are the need for the biobanking of glioma patients’ blood and the need for standardized methods pursued with great accuracy to make these results easily reproducible. The clinical utility of bloodstream-based biomarker research should be investigated further. Further studies are needed to determine if an alternative biofluid could be used in addition to bloodstream tests, such as CSF, urine, or saliva.

Moreover, the opportunity to group different detection methods should be considered to identify tumor-specific characteristics, such as tumor origin, grading, and malignancy features; these traits should also be related to the patient prognosis. In addition, frequent evaluations should assess tumor response during follow-up.

The characteristics above have already been evaluated for each biomarker, but their cumulative analysis could represent a significant problem, given the lack of funds, technicians, machinery, and technological tools.

## 5. Conclusions

The complexity and malignancy of GBM lead the scientific world on a critical search for the newest therapies and precise techniques to improve the quality of life and OS in affected patients. Still, none of them has been validated until now. Studies included in the present literature review have shown the increasing desire to find innovative methods to analyze GMB in its entirety. As a result of these efforts, liquid biopsy is advocated as becoming part of every clinical setting. By creating a more specific and worldwide-connected biobank, the validation of liquid biopsy in clinical practice can be validated. Moreover, liquid biopsy would be considered a valid alternative to a surgical biopsy, thus acquiring the dignity of a prognostic tool.

## Figures and Tables

**Table 1:** Overview of all included studies reporting the role of liquid biopsy in clinical setting and sensitivity/specificity of the biomarkers studied.

Author, Year	Type of Study	Patients (*n*)	Biomarker	Sample	Detection Method	Role	Sensitivity	Specificity
Olioso D. et al., 2021 [20]	Prospective study	52 GBM 5 anaplastic astrocytoma	miR-21 miR-222 miR-124-3p	serum	qRT-PCR	- Discrimination between stable and progressed patients - Prediction of poorer prognosis - Prediction of response to therapy	- miR-21 AUC 0.52, miR-222 AUC 0.51 (T1); miR-21 AUC 0.53, miR-222 AUC 0.58 (T2) - miR-124-3p AUC 0.71 - miR-21 AUC 0.78 (T3)	
Swellam M. et al., 2021 [21]	Prospective study	25 GBM 20 HC	miR-17-5p miR-125b miR-221	blood	qRT-PCR	- Diagnostic - Prognostic - Impact of miRs expression on response to treatment	50.5% 52.9% 76.5%	100% 100% 100%
Palande V. et al., 2021 [22]	Prospective study	25 GBM 25 HC	cfDNA	plasma	NGS	- Diagnostic - Monitoring - Targeted therapy	80%	90%
Zhang H. et al., 2019 [23]	Prospective study	95 GBM 60 HC	miR-100	blood	qRT-PCR	- Diagnostic	77.89%	83.33%
Swellam M. et al., 2019 [24]	Prospective study	20 GBM, 20 HC	miR-221 miR-222	Serum	qRT-PCR	- Diagnostic - Prognostic	90% 90%	90% 85%
Manda SV. et al., 2018 [25]	Prospective study	96 high-grade gliomas 50 HC	EGFRvIII RNA in serum-derived EVs	Serum	PCR	- Diagnostic	81.58%	79.31%
Figueroa JM. et al., 2017 [26]	Prospective study	71	wtEGFR and EGFRvIII RNA in CSF-derived EVs.	CSF	PCR	- Diagnostic and monitoring	61%	98%

Abbreviations: HC: healthy controls; GBM: glioblastoma; cfDNA: circulating free DNA; EGFRvIII: epithelial growth factor receptor variant III; CSF: cerebrospinal fluid; qRT-PCR: quantitative real-time—polymerase chain reaction; NGS: next-generation sequencing

## Data Availability

Not applicable.
